# Human Urine Proteomics: Analytical Techniques and Clinical Applications in Renal Diseases

**DOI:** 10.1155/2015/782798

**Published:** 2015-11-29

**Authors:** Shiva Kalantari, Ameneh Jafari, Raheleh Moradpoor, Elmira Ghasemi, Ensieh Khalkhal

**Affiliations:** ^1^Chronic Kidney Disease Research Center, Labbafinejad Hospital, Shahid Beheshti University of Medical Sciences, Tehran, Iran; ^2^Department of Basic Science, Faculty of Paramedical Sciences, Shahid Beheshti University of Medical Sciences, Tehran, Iran

## Abstract

Urine has been in the center of attention among scientists of clinical proteomics in the past decade, because it is valuable source of proteins and peptides with a relative stable composition and easy to collect in large and repeated quantities with a noninvasive procedure. In this review, we discuss technical aspects of urinary proteomics in detail, including sample preparation, proteomic technologies, and their advantage and disadvantages. Several recent experiments are presented which applied urinary proteome for biomarker discovery in renal diseases including diabetic nephropathy, immunoglobulin A (IgA) nephropathy, focal segmental glomerulosclerosis, lupus nephritis, membranous nephropathy, and acute kidney injury. In addition, several available databases in urinary proteomics are also briefly introduced.

## 1. Introduction

Clinical samples such as tissues and bio fluids (e.g., serum, plasma, urine, and saliva) are undoubtedly valuable sources in biomarker discovery studies for diagnostic proposes. The protein content complexity is the first issue in handling and analysis of such samples. As urine, which contains approximately 2000 proteins [1, 2] and is a less complex sample than plasma which contain more than 10,000 core proteins [3], could be collected in a noninvasive and unrestricted way, it is a preferable resource for investigation of a broad spectrum of diseases. Moreover, the protein composition and fragmentation of urine are relatively stable in comparison with other biofluids such as plasma or serum which are prone to proteolytic degradation during and after sampling [4].

As urine is the consequence of blood filtration and also contained all secreted proteins from tubules and kidney specific cells [5–10], it thus has been studied for investigating the pathological process of systemic as well as renal diseases [11]. Serum proteins are filtered based on their sizes and charges at the glomeruli [12] while reabsorption of abundant serum proteins such as albumin, immunoglobulin light chain, transferrin, vitamin D binding protein, myoglobin, and receptor-associated protein occurs in proximal renal tubules mainly by endocytic receptors, megalin, and cubilin [13–16]. Thus, protein concentration in normal donor urine is very low (less than 100 mg/L when urine output is 1.5 L/day), and normal protein excretion is less than 150 mg/day. This is about a factor 1000 less compared with other body fluids such as plasma. Excretion of more than 150 mg/day protein is defined as proteinuria and is indicative of glomerular or reabsorption dysfunction.

Urine proteomics studies account for several barriers such as low concentration of total protein, high concentration of salts and other ingredients that hinder protein separation [17], and high dynamics of changes in urine composition between different seasons of the material collection. Due to differences in sensitivity and availability of various proteomic techniques, considerable efforts have been made to find a suitable method to analyze the expression of specific urine marker proteins [18].

Urine proteomics is a powerful platform to identify urinary excreted proteins and peptides in different stages of disease or therapy and to determine their quantity, functions, and interactions [19]. Therefore, mechanism of the disease and novel therapeutic targets could be suggested by proteomic approaches. In this review we focus on technical aspects of analyzing urine proteome; application of this platform in clinic and also several urine proteome databases are reviewed.

## 2. Normal Human Urinary Proteome

Normal human urine contains a significant amount of peptides and protein. In 2004 nearly 1400 protein spots were separated by using two-dimensional gel electrophoresis (2DE) and 150 distinct proteins from 420 spots were identified by mass spectrometry [8]. This number of identified urinary proteins increased significantly to around 1534 in 2006 by combining one-dimensional gel electrophoresis and reverse phase liquid chromatography coupled to mass-spectrometry [1]. To date, over 2000 proteins in total are estimated in normal human urine of which 1823 proteins were identified by Marimuthu et al. in 2011 [20]. Identified proteins by Marimuthu et al. were ~300 greater than Adachi's report and hence could serve as a comprehensive reference list for future studies. Some of the urinary proteins have greater experimental molecular weights than the theoretically possible from amino acid sequence alone, indicating the presence of posttranslational modifications. 225 N-glycoproteins [10] and 31 phosphoproteins [21] were identified in normal human urinary proteome. Normal human urine contains exosomes, small vesicles with diameters less than 100 nm that are secreted from renal epithelial cells.

Exosomes contain a number of disease related proteins. There are 1132 proteins in urinary exosomes including 14 phosphoproteins [22]. It is known that the urinary proteome differs between healthy individuals, particularly between men and women. In addition to the interindividual differences, the urinary proteome from the same individual varies at different time points due to the effect of exercise, diet, lifestyle, and other factors. The urinary proteome changes significantly over time. Urine collected in the morning contained more proteins than in the afternoon and evening. In addition, the interday proteome variation was observed to be greater than the variation at different time points in the same day [23].

## 3. Urine Preparation

Optimization of sample preparation is a necessary first step for urinary proteome analysis and sample desalting; that is, using precipitation or dialysis is often required. Several protocols that are used to isolate urinary proteins were employed using precipitation, lyophilisation, ultracentrifugation, and centrifugal filtration. Acetone precipitates more acidic and hydrophilic proteins. Ultracentrifugation fractionates more basic, hydrophobic, and membrane proteins. Organic solvents (90% ethanol and 10% acetic acid) precipitate urinary proteins in the highest recovery rate. The ACN-precipitated urine sample produced the greatest number of spots on a two-dimensional (2D) gel, whereas the acetic-precipitated sample yielded the smallest number of spots [24]. The dialysis of urine proteins and concentration by lyophilisation without fractionation significantly improve reproducibility and resolution and are probably able to reflect total urinary proteins on 2D gels. In addition, the removal of albumin from the urine helps to identify the low abundant proteins [25]. Two main variables have been analyzed in urine preparation methods: quantity (protein recovery yield) and quality (2D spot patterns or proteomic profiles). The conclusions reached are that there is no single perfect protocol that can be used to examine the entire urinary proteome since each method has both advantages and disadvantages in comparison with the others. A combination of several sample preparation methods is required to obtain the greatest amount of quantitative and qualitative information [24].

## 4. Urine Proteome Enrichment

Due to the fact that disease specific biomarkers are likely in the low abundant fraction of proteome which are masked by high abundant proteins, enrichment strategies may increase the chance of capturing these potential biomarkers.

Depletion of high abundant proteins using chromatographic based enrichment methods or commercial kits specialized for urine samples are available strategies for enrichment.

Several high abundant proteins (e.g., albumin, transferrin, haptoglobin, immunoglobulin G, immunoglobulin A, and alpha-1 antitrypsin) could be removed using antibody-based affinity depletion approaches (e.g., multiple affinity removal system (MARS)) that are able to be coupled with either 2DE or LC-MS analysis [26, 27]. Enrichment of low abundant and depletion of high abundant proteins could be combined in peptide ligand library approach. In this method a diverse library of peptides immobilized on a solid-phase chromatographic matrix interact with the specific recognition site of proteins. Captured proteins, therefore, are included in the analysis procedure while other proteins without suitable interaction are washed out and excluded. Saturation of peptides for high abundant proteins leads to decrease in their concentration in the final eluent. In addition, concentrated low abundant proteins on their specific ligands result in decreasing the dynamic range of proteins in the specimen [28]. The success of this method depends on using high amounts of initial material; otherwise, the enriched profile would be similar to the nonenriched original profile [29]. Chromatography techniques such as ion-exchange, size-exclusion, and affinity chromatography are widely used for enrichment or depletion purposes; however, they are also applicable for fractionation and separation prior to MS (see Section 5.2).

Charge-charge interaction between proteins of the sample and charged column is the base of capturing subproteome in ion-exchange chromatography. In anion exchange chromatography, negatively charged proteins or peptides interact with positively charged column and gradually eluted off the column by a mobile phase with gradient of salt solution. Majority of bound proteins with negative charge are eluted at the highest ionic strength [30]. Lu et al. enriched low-abundance proteins in urine specimen using this technique and observed improved number of identification in 2DE map of enriched samples in comparison with the map of nonenriched samples [30].

In contrast to anion exchange, charge of stationary phase in strong cation exchange chromatography (SCX) becomes negative in aqueous solution and therefore interacts with strongly basic analytes. To elute the analytes, column is then washed with a solvent with pH gradient. The most cationic proteins/peptides would elute off the column at higher pH. Therefore, this technique has been introduced as a suitable method for phosphoproteome enrichment [31]. Thongboonkerd et al. showed application of SCX for enrichment of the basic/cationic urinary proteome [32].

Commercial protein depletion kits are currently available that some of them have been optimized for proteomic studies on urine samples (e.g., UPCK urine protein enrichment/concentration kit). According to the literature using these depletion kits could improve the number of low-abundance proteins identified in urine up to 2.5-fold [33].

Selection of the enrichment strategy depends on the aim and specific study requirements.

## 5. Techniques for Urinary Proteomic Studies

Common techniques for urinary proteome analysis are two-dimensional gel electrophoresis followed by mass spectrometry (2DE-MS), liquid chromatography coupled to mass spectrometry (LC-MS), surface enhanced laser desorption/ionization coupled to mass spectrometry (SELDI-TOF), and capillary electrophoresis coupled to mass spectrometry (CE-MS) and protein microarrays [34].

### 5.1. DE-MS

Two-dimensional gel electrophoresis is a robust and widely used method for protein separation. In this technique proteins are separated based on their isoelectric point and molecular mass, visualized, and semiquantified by staining [25]. Advantages of 2DE include ability to detect relatively large molecules, estimate molecular weight and pI of proteins, and investigate posttranslational modifications [35]. Common limitations of 2DE technique are requirements for high protein amount, lack of automation, loss of extremely acidic (pH < 3) or basic proteins (pH > 10), lack of detection for large (Mr > 150 kD) and small (Mr < 10 kD) proteins and hydrophobic proteins, and lack of reproducibility, low-throughput capacity, and narrow dynamic range [36, 37]. A few of these limitations have been granted by some technical improvements. Development of two-dimensional difference gel electrophoresis (2D-DIGE) ameliorated problem of low-throughput capacity improve the quantification accuracy and statistical confidence by use of internal standard and pooling samples labeled with different fluorescent dyes (Cy2, Cy3, and Cy5) [38]. Blue native technique was developed in order to specify investigations on hydrophobic proteins, protein complexes, and protein-protein interactions on the gel matrix [39]. Blue native technique separates proteins based on their molecular weights on two dimensions. The reproducibility problem has been minimized using precast gels with multirun gel tanks which are capable of running several electrophoretic gels simultaneously. Furthermore, staining gels with different fluorescent dyes postelectrophoretically increase the sensitivity of detection spots. In case of study the urinary proteins by 2DE, desalting by chromatographic columns or filters is recommended to obtain well separated spots on the gel. Despite improvement in 2DE technique, lack of automation and time consuming steps (approximately 3 days for obtaining a 2DE pattern) lead to decrease inclination of researchers for applying this technique in their recent experiments.

### 5.2. LC-MS

Liquid chromatography (LC) is a high resolution separation method that employs one or more inherent characteristics of a protein, its mass, isoelectric point, hydrophobicity, or biospecificity [40]. This method separates large amounts of analytes (protein/peptides) on HPLC column or small amount of analytes (peptides) on a capillary LC column with high sensitivity and can be automated [41]. LC/MS can identify low-abundance and hydrophobic proteins not seen by 2DE and thus is considered a complementary method for 2DE in proteomics 2D liquid phase fractionation and can be used for in-depth analysis of body fluids such as urine [42]. A combination of gel and different steps of gel-free techniques (i.e., sequential separation using different matrices in two or more independent steps) is used for a better separation and referred to as Mud PIT. Strong cation exchange column (SCX) is a good choice for separate urinary peptides before injection to reverse phase columns coupled to MS. The retention times of many urinary compounds including organic acids and bile salts overlap with peptides in reverse phase LC which can be removed using SCX column [43]. Recently, weak anion exchange columns are used for fractionation and enrichment of low abundant proteins excreted in the urine prior to 2DE [44]. This combination method has potential for identification of low abundant biomarkers but still has challenges for identification of isoforms and PTMs. Affinity chromatography columns before LC-MS runs are the other alternative methods for capturing subproteomes from the urine such as glycoproteome and phosphoproteome [22, 45]. LC technique can be coupled with diverse types of mass spectrometry instruments that affect the accuracy and confidence of identification and quantification.

The high resolution instruments such as the Fourier transform ion cyclotron resonance (FTICR) and orbitrap or hybrid and tribrid instruments such as Q-exactive (hybrid of quadrupole and orbitrap) and orbitrap Fusion (tribrid of quadrupole, orbitrap, and linear ion trap) may couple to LC for clinical sample analysis including urine. Kalantari et al. and Adachi et al. analyzed urine samples from IgA nephropathic patients and normal human urine, respectively, using LC coupled with high resolution MS instruments [1, 46]. Proteins separated by LC could be quantified with the labeling (e.g., stable isotope affinity tag and isobaric tags) and label-free techniques [41]. Quantification of proteins or peptides in large scale is possible only by gel-free MS based methods which is considered an advantage for these techniques. As LC-MS is time-consuming and sensitive towards interfering compounds (e.g., salts) and precipitation of analytes on column materials, it is not applicable yet for routine clinical diagnostic tests [48].

### 5.3. SELDI-TOF

Surface-enhanced laser desorption/ionization time of flight (SELDI-TOF) technology uses protein-chip arrays with different properties on the surface (hydrophilic or hydrophobic materials, cationic or anionic matrices, lectin, or antibody affinity reagents) coupled with a TOF mass spectrometer [36]. Proteins or peptides of interest are bound to the active surface depending on the surface property while unwanted unbound proteins are washed away with an appropriate solvent or buffer [36, 49]. It is a widely used method in clinical proteomics that can detect different protein expression patterns of body fluid and tissue specimens between patients and healthy subjects and thus is a powerful tool for biomarker discovery [50]. SELDI-TOF is a high throughput and easy to use technique that can be automated. In addition, a low sample volume (<10 *μ*L) without prior concentration or precipitation of proteins is required [42, 51]. However, it has some limitations such as difficulties in standardization since the proteome profiles generated by SELDI are influenced by many factors such as the type of surface coating, pH, salt condition, and protein concentration. Other limitations of SELDI are lack of reproducibility, restriction to selected proteins, low-resolution mass spectrometer, and lack of a sequence based identification of the resolved peaks [48, 52, 53].

### 5.4. CE-MS

Capillary electrophoresis coupled to MS represents ideal analytical technique for different omics approaches that provides high resolution protein separation based on differential migration through a buffer-filled capillary column in an electrical field (300 to 500 V/cm) [54, 55]. CE can be coupled either with MALDI (off-line) or ESI (on-line). While data analysis of CE-MALDI is more sight forward, signal suppression and variability results from matrix effect as well as loss of resolution are considered challenges in this method and thus coupling with ESI is preferred [56]. Though CE is capable of being coupled with any type of MS instrument, ESI-FT-ICR is the most applicable one for identification of proteins and peptides disease biomarkers in urine [47, 57]. CE-MS offers several advantages including fast and high efficient separation, selectivity, sensitivity, low cost, absence of buffer gradients, capability of fast reconditioning with NaOH, and insensitivity towards precipitating proteins peptides, lipids, and other compounds that often interfere with LC-separation [51, 58].

This method is especially applicable for analysis of the low molecular weight (<20 kDa) molecules. Its disadvantages are limited capacity to separate high molecular weight proteins (>20 kDa) and low-abundance proteins, lack of reproducibility and robustness, and small sample loading capacity [53, 55].

### 5.5. Protein Microarrays

Protein microarrays (or protein chips) are miniaturized solid-phase ligand-binding assay systems using immobilized antibodies or antigens on a support surface, generally a slide or membrane [59]. According to different features, such as content, surface, or detection system, there are many types of protein microarrays [60]. Main advantages of protein microarrays include high-throughput; sensitivity and discovery of low molecular weight markers make them an ideal approach for urinary proteomics. However, microarrays have several limitations such as requirement for a highly specific probe for each analyte, low density coverage that allows detection of only a few proteins, variable specificity, and lack of detection of posttranslational modifications [25, 61].

## 6. Advances in Urine Proteomic Research

Over the last two decades, advances in proteomic tools have been impressive. “Microfluidic chip CE (MC-CE) device,” a recent analyzing tool for biomarker discovery, is one of the precious advances that enabled profiling of urinary markers with adjustable on-chip sample dilution [62]. This device is specially suitable for detection of urine anionic biomarkers and has high separation efficiency and sensitivity in analyte detection. It is composed of two units: sample dilution and on-chip CE separation [63]. In this device sequence dilution and separation are applicable by magnetic fluid activated valves. Recent studies on urine samples using microfluidic devices have been reviewed by Lin et al. [64].

Filter aided sample preparation (FASP), a method of detergent depletion, is one of the effective recent technique in shotgun proteomic analysis [65]. This method benefits from the advantages of both in-gel and in-solution digestion and has high protein coverage [66].

Low volume of sample is required and feasibility of digesting protein mixtures directly on the filter membrane is the most important advantages of this method.

96-well based parallel FASP is a robust method in urine proteomics which is cost-effective, repeatable, and efficient. Wiśniewski et al. and Yu et al. [65, 66] have applied this technique successfully for analyzing urine samples.

Advances in quantification methods provide more information from peptides and proteins in biological samples including urine. In label-free quantification the main goal is to develop the software with capability of normalization and reduced errors originated from instrument and loading samples. “Quanti” is a right example of recently developed software which is capable of relatively accurate label-free quantification of proteins with correction of responses to instrumental fluctuation [67]. This software has been applied in several studies on urine proteomics [46, 68–71]. MaxLFQ is also newly developed label-free software that can handle very large experiments, uses delayed normalization which makes it compatible with different separation procedures, and extracts the maximum ratio information from peptide signals [72]. Other label-free software programs and advantages and limitations of this technique have been reviewed elsewhere [73, 74].

Labeling techniques for quantification seems to be less improved during recent years in competition with label-free quantification techniques. However, isobaric tags for relative and absolute quantification (iTRAQ) as a versatile labeling technique which developed early in 2000s decade are still popular and frequently used specially in urine proteomics studies [75].

## 7. The Use of Urine in Clinical Proteomics

A major challenge in clinical proteomics is the identification of reliable biomarkers that help early diagnosis of disease and contribute to the development of personalized medicine. As 70% of urinary proteins stem from kidney and urinary tract and 30% originate from the other organs that are secreted into the blood circulation, study of the urinary proteomics could be useful in better understanding of pathophysiological mechanisms and the discovery novel biomarkers and therapeutic targets of kidney and nonkidney diseases [76]. Anderson et al. reported the identification of several proteins as urinary biomarkers in 1979. The explanation of disease-specific biomarkers in the urine is complicated by significant changes in urinary proteome during the day under the influence of some factors such as exercise, variations in the diet, and circadian rhythms. Simply comparing the proteome urine of patients with a particular disease with healthy individuals to distinguish between different diseases with similar symptoms is inadequate. Since the variability of conditions and irrelevant differences between healthy people and target diseased group are inevitably large and uncontrolled, comparison of one or more symptomatic similar diseases but different in etiology and severity simultaneously as controls is recommended for biomarker discovery. Thus, careful experimental design is the key to success in biomarker studies. Another issue is the correct matching of case and control groups. For instance, since some diseases such as cancer and chronic kidney disease usually occur in middle-aged and elderly people, urine samples from young volunteers are not appropriate controls for studying these diseases. Urine proteomics, if observing the careful study design, is the promising platform for identification of early detection and noninvasive biomarkers in the new era of modern medicine.

Despite large number of studies published in the past decades using proteomic tools in biomarker discovery field, still no reliable, specific, sensitive biomarker is available. A general shortcoming of biomarker research is lack of reproducibility and effect of unknown factors originates from dark side of disease pathogenesis which we do not have enough knowledge about and therefore could not be controlled. This concern will be fade in the future by advances in preparation methods, separation procedure, and extended databases. In addition, following a comprehensive workflow in clinical proteomics from initial discovery to translation into a clinically useful assay would help a biomarker to be meaningful and applicable in the clinic. Mischak et al. defined six steps for this workflow: (I) initial identification and verification, (II) evaluation the results by a knowledgeable independent panel of experts, (III) evaluation in suitable biobank samples or newly collected samples, (IV) evaluation in clinical trial, (V) implementation in clinical practice, and (VI) proving the cost-effectiveness of validated biomarker [77].

Another important point that should be considered in the context of biomarker implementation in clinics is the use of a panel of biomarkers instead of a single biomarker. A panel of biomarkers can lead to increase sensitivity and accuracy of assays.

### 7.1. Urinary Biomarkers in Renal Disease

#### 7.1.1. Diabetic Nephropathy

Diabetic nephropathy (DN) is a serious complication of diabetes with a complex etiology that involves up to 40% of diabetic patients [39, 43]. DN is diagnosed by excretion of 30–300 mg protein in the urine per 24 hours (microalbuminuria) which can progress to macroalbuminuria (>300 mg/24 h) and/or changes in serum creatinine indicating decline in the glomerular filtration rate. The histological presentations of DN are loss of podocytes, thickness in glomerular basement membrane, proliferation of mesangial cells, and tubule interstitial fibrosis [44]. The current diagnostic tool for DN is biopsy that is invasive and not recommended for all suspicious cases due to its consequences. Urinary diagnostic biomarkers are promising noninvasive diagnostic molecules complementary to biopsy that will enter into the clinic in the near future. These diagnostic molecules can either promote the accurate diagnosis or decision for a more effective treatment.

Microalbuminuria (MA) is important for early diagnosis of DN and is the best predictor of DN available in the clinic [46]. However, several studies have shown that only a subset of patients with MA progress to proteinuria [47, 56]. Moreover, many individuals with type 1 diabetes have already experienced early renal function decline before or accidental with the onset of MA. So, it seems that MA may be an inadequate early diagnostic biomarker of DN (however, it may be appropriate for diagnosis of advanced DN) and discovery of new sensitive and specific markers by proteomic techniques is necessary [76]. In addition, some of the suggested biomarkers for DN have been achieved on samples from patients whose disease was diagnosed by clinical criteria and not confirmed by biopsy which is highlighted necessity of more accurate studies on biomarker discovery for DN. The study of Soldatos and Cooper [44] was a well-designed pioneer pilot study for profiling the urine proteome of DN patients using SELDI-TOF for discovery of early diagnostic markers. They could identify a 12-peak urine protein signature in type 2 diabetic patients compared with healthy controls with 93% sensitivity and 86% specificity. Interestingly, patients in their study were normalbuminuric (albumin-to-creatinine ratio < 30 mg/g) without renal dysfunction. In a case-control study Rossing using high-resolution two-dimensional gel electrophoresis separation and protein identifications by MALDI-TOF-MS and LC-MS/MS analysis [43] reported correlation between level of a set of proteins, in particular Tamm-Horse fall urinary glycoprotein (THP) and zinc-*α*-2 glycoprotein (ZA2G), and the state of diabetic progressing in diabetic patients type 1. Jim et al. using ELISA indicated that nephrinuria is detectable in all of type 2 diabetic patients with macroalbuminuria and microalbuminuria and thus nephrine has potential to be a new early biomarker of DN [78]. Capillary electrophoresis-coupled mass spectrometry was used to profile urine proteome of DN patients in a longitudinal study. Altered content of collagen fragments was suggested as a potential early detection biomarker which occurs 3–5 years before onset of macroalbuminuria [79].

The most recent report of protein biomarkers for the stages of DN using iTRAQ suggested a diminished excretion of pancreatic amylase and deoxyribonuclease I [80]. Some of the novel urinary biomarkers for DN reporting in the last two years are tabulated in Table 1.

As biopsy is not performed in all patients suffering from diabetes (due to its invasiveness), DN is diagnosed based on clinical manifestations in some cases and, therefore, treated based on available guideline for DN. Nevertheless, interestingly, kidney injury in some of diabetic patients would not be a result of diabetic nephropathy and other kidney diseases such as membranous nephropathy or focal segmental glomerulosclerosis might also occur in diabetic patients which could not be diagnosed only by clinical manifestations. Therefore, biomarkers sensitive and specific for DN are undoubtedly needed as a noninvasive alternative of kidney biopsy.

#### 7.1.2. IgA Nephropathy

IgA nephropathy (IgAN) is the most common type of glomerulonephritis worldwide that is characterized at biopsy by histological features including mesangial immunodeposits of IgA1 (by immune fluorescence) in association with C3 and IgG or IgM or both [81, 82]. The clinical presentation of IgAN is variable and could be a spectrum of clinical features from asymptomatic hematuria to rapidly progressive glomerulonephritis. Urinary excretion of relevant proteins such as immunoglobulin, cytokines, and complement factor suggest a promising new possibility in the search for noninvasive diagnostic markers in patients with IgAN.

SELDI-TOF as one of the powerful techniques in urinary proteomics was used by some research groups for biomarker discovery of IgAN. Rocchetti et al. analyzed urine proteome of 49 IgA nephropathy patients, 42 CKD (chronic kidney disease) patients, and 40 healthy individuals using this technique followed by MALDI-TOF for identification of differentially proteins and reported Perlecan laminin G-like 3 peptide and Ig*κ* light chains as indicator of disease activity [83].

Julian et al. applied CE-MS for detection of urinary polypeptide biomarkers differentiated patients with IgA nephropathy from other renal diseases including diabetic nephropathy, lupus nephritis, hypertensive renal disease, acute vasculitis with nephritis, and amyloidosis and from subjects with a functional renal allograft [84]. Some of these markers were identified by top-down MS/MS approach (e.g., alpha-1-antitrypsin, collagen type III alpha-1 chain, and uromodulin). In addition, they defined a panel of biomarkers named “Renal Damage Pattern” by comparing pattern obtained from healthy controls and several renal diseases. These markers then correlated with IgA nephropathy pattern using SDS-PAGE/western blotting.

Zhao et al. for first time used SILAC-labeled mouse serum as internal standard for human and urine proteome analysis by IEF-LC-MS/MS. They compared urine from IgAN patients treated and untreated and reported fifty-three peptides that were different between two groups. The authors could find novel candidates like ApoA1 and insulin-like growth factor-binding protein 7 (IGFBP7) for IgAN by this quantitation strategy [85]. Kalantari et al. in a pilot study on urine samples from 13 patients with IgA attempted to find a correlation between proteome data and pathological presentation used for classification of IgAN in biopsy through a high resolution mass spectrometry [46]. They performed two independent proteomic procedures (nano-LC-MS/MS and GeLC-MS/MS) and reported a panel of candidates as prognostic markers including afamin, leucine-rich alpha-2-glycoprotein, ceruloplasmin, alpha-1-microgolbulin, hemopexin, apolipoprotein A-I, complement C3, vitamin D-binding protein, beta-2-microglobulin, and retinol-binding protein 4. The recent study on IgAN with a cohort of 30 patients and 30 controls suggested 18 differential urinary proteins separated and identified by IEF/LC-MS/MS [86]. Most of these reported candidates were complement components, coagulation factors, and extracellular and intracellular matrix and transmembrane.  Table 2 shows some of the recent (last 3 years) reported urinary markers for IgAN.

#### 7.1.3. Focal Segmental Glomerulosclerosis

Focal segmental glomerulosclerosis (FSGS) is a glomerular podocytopathy characterized by massive proteinuria as clinical manifestation and glomerular scaring as histological feature [87, 88]. hypoalbuminemia, hypercholesterolemia, and peripheral edema are associated with proteinuria and considered also clinical manifestations [87]. It is a complex disease categorized as primary (~80%) or secondary (~20%) based on the etiology. Diffuse foot process effacement of podocytes is mostly associated with primary form detected by electron microscopy [88]. Increasing frequency of FSGS in the past 20 years with 50% ESRD rate during 5–8 years from the time of diagnosis in resistant patients to therapy [88, 89] indicates the need of biomarkers for early diagnosis and prediction of responsiveness. Molecular biomarkers are helpful not only in diagnosis but even in understanding the pathogenesis and disease mechanism.

Shui et al. performed a classic proteomic study on urinary biomarkers FSGS mouse model [90]. Serial urine samples was collected on days 0, 4, 7, 11, 15, and 20 and analyzed by two-dimensional electrophoresis followed by MALDI-TOF-MS. They identified 37 proteins changed during the disease course and confirmed few of them via western blot. Collagen IV fragment, glutathione S-transferase, and E-cadherin were suggested as candidates for disease progression. The other candidates for early diagnosis identified in their study are tabulated in Table 3.

Wang et al. also studied biomarkers to distinguish between adriamycin nephropathy and Thy1.1 glomerulonephritis in the rat model [10]. Urine proteome was precipitated by acetone and, after protein concentration determination, equal urine proteome from each five sample pooled in four groups and subjected to lectin enrichment. LC-MS/MS analysis on eluted glycoproteome followed by quantification using spectral counting approach resulted in 46 differential proteins including Ig lambda-2 chain C region, protein YIPF3, hemopexin precursor, and 35 other proteins with different direction of changes and serum albumin precursor, isoform 1 of serotransferrin precursor, alpha-1-antiproteinase precursor, T-kininogen 1 precursor, trefoil factor 3 precursor, superoxide dismutase, and urinary protein 1 precursor with similar direction of changes.

SELDI technique was applied by Woroniecki et al. to identify different pattern in urine proteome of healthy control and steroid resistant (SRNS) and steroid sensitive (SSNS) patients with nephrotic syndrome such as MCD and FSGS [91]. Predictive models were constructed by supervised algorithm to differentiate between control and diseased (combination of SSNS and SRNS) and between SSNS and SRNS. Generated model for responsiveness prediction had 100% accuracy and resulted in a differential protein of mass of 4,144 daltons with significant changes as the most important classifier.

Zhao et al. recently performed a study on urine biomarkers which reflect dynamic changes during disease course, analyzed by UPLC coupled with triple-TOF-MS, quantified by label-free quantification, and confirmed by western blot [92]. They examined six stages in ADR-induced rats. Relative abundance of twelve proteins showed an overall increasing trend while nine proteins shared an overall decreasing trend. Fetuin-B and B2-microglobulin changed at the early stage. These markers were further investigated to find human orthologues. Confirmed suggested candidates are shown in Table 3.

A few studies with proteomic approaches have been performed recently for identification biomarkers specially in the urine samples of FSGS patients. In several studies FSGS subjects were not the main target and were considered the control disease. Additional longitudinal studies are required to determine more valuable noninvasive biomarkers for FSGS in the context of early detection, treatment response, and prognosis.

#### 7.1.4. Lupus Nephritis

Lupus nephritis (LN), a common consequence of systemic lupus erythematous (SLE), is associated with significant mortality and morbidity. LN is classified to six classes based on histologic presentations as well as two score indices: activity and chronicity. Currently, renal biopsy beside clinical presentations is used for diagnosis of LN. The challenging part is the treatment which differs totally based on the class and activity and chronicity indices. Therefore, careful diagnosis is critical for treatment. Moreover, current markers for LN such as proteinuria, serum creatinine level, creatinine clearance, complement levels, anti-dsDNA, and antinuclear antibodies are not sensitive or specific enough and diagnosis based on biopsy is sometimes impossible to perform invasively. Therefore, there is a need for some biomarkers which correlate with renal activity to be used for diagnosis of the disease class, early detection before renal failure, and prediction of the prognosis and response. Urinary biomarkers detected by proteomic tools are appropriate candidates to fulfill these issues.

Urinary VCAM-1, CXCL16, P-selectin, and TNFR-1 are diagnostic candidates detected and validated by ELISA [93, 94]. Urinary monocyte chemotactic protein (MCP-1) and TWEAK level have been suggested as an indicator of disease activity also using ELISA [95, 96].

Detection of transferrin, ceruloplasmin, *α*1-acid-glycoprotein (AGP), lipocalin-type prostaglandin D-synthetase (LPDGS), albumin, and albumin-related fragments in the urine of pediatric lupus nephritis patients using MALDI-TOF is one of the outstanding studies performed by Suzuki et al. [94]. Important points of this report were correlation of these candidates with disease activity and increase in level of some of them (such as transferrin, AGP, and L-PDGS) before clinical presentations of disease flare. Other candidates which were reported as predictor marker of flare were reported later by Lee et al. using SELDI-TOF [97]. They detected increased excretion of hepcidin 20 and albumin fragment (N-terminal region), four months before the flare, and decrease of excretion of hepcidin 25 at the flare time. This technique was also used by Mosley et al. for discrimination between patients with active and inactive forms of lupus nephritis [98]. The proteins with masses 3340 and 3980 were differentially excreted between these two forms which were not further identified.

A classical 2D electrophoresis followed by MALDI-TOF by Oates et al. showed a list of candidates for diagnosis of the class and chronicity of which highest sensitivity belonged to a-1 acid glycoprotein [99].

#### 7.1.5. Membranous Nephropathy

Membranous nephropathy (MN) is categorized as one of the most common causes of primary glomerular diseases especially in adults [100]. MN is a kidney-specific autoimmune disease in which circulating autoantibodies (such as IgG4) bind to intrinsic antigen on glomerular podocytes (i.e., M-type phospholipase A2 receptor 1in primary form of MN), form immune complex, and deposit in subepithelial area of the basement membrane [101]. Therefore, thickening of glomerular basement membrane due to immune complex depositions is a histologic hallmark under the light microscopy [102]. As other renal diseases, the main reason for scientists' interest in discovering urinary biomarkers for MN is invasiveness of kidney biopsy and its potential risk of serious complications.

A classic proteomic study on urine samples collected from animal model of passive Heymann nephritis (PHN), which mimics human membranous nephropathy, using 2D-PAGE and SYPRO Ruby staining followed by MALDI-TOF-MS showed a panel of potential biomarkers [103]. Serial urine samples in 6 different time points after the injection with anti-Fx1A were collected. Signaling pathways, glomerular trafficking, and controlling the glomerular permeability altered significantly in the disease course. Despite its good design, lack of enough number of technical replicate could be considered a weak point for their study.

Recent study on urine microvesicles obtained from idiopathic membranous nephropathy (iMN), focal segmental glomerulosclerosis (FSGS) patients, and healthy controls via iTRAQ labeling system revealed twofold increases in lysosome membrane protein-2 (LIMP-2) excretion in iMN group [104]. The pooled iTRAQ8plex samples were fractionated using strong cation exchange (SCX) and reverse phase (RP) columns and analyzed with LTQ orbitrap mass spectrometer. Glomerular expression of LIMP-2 was further investigated using immunofluorescence staining which was consistent with proteomic results.

Apart from importance of biomarkers in prompt diagnosis of this disease because of high rate of ESRD in MN patients (~40%) [105], identification of biomarkers predictive of responsiveness to treatment is also critical. To our knowledge, no investigation using routine proteomic tools (i.e., 2DE or LC coupled to mass spectrometry) is still available; however, Irazabal et al. examined the relationship between a panel of known biomarkers with responsiveness of MN patients to rituximab with western blot and ELISA [106]. They suggested urinary IgG (mg/24 h) as a significant predictor for proteinuria changes at first year of therapy while fractional excretion of IgG, urinary alpha 1 microglobulin (U*α*1M) (mg/24 h), and urinary retinol binding protein (URBP) (*μ*g/24 h) were predictor of the response at 12 months but not at 24 months.

Applying high-throughput proteomic tools in identification of the predictive biomarkers for MN is a promising approach which needs more effort. Some of the studies on urine proteins aiming at biomarker discovery are tabulated in Table 4.

#### 7.1.6. Acute Kidney Injury

Acute kidney injury (AKI) is a complicated condition encompassing wide range of clinical manifestations (e.g., elevated serum creatinine and anuric renal failure) [107]. This disorder has high rate of mortality and morbidity which mostly affected critically ill patients (e.g., patients admitted to ICU) and associated with a sudden fail in renal function [108]. The markers that are currently used in clinic such as serum creatinine and decline in GFR are detected late after kidney injury. Therefore, lack of early markers leading to delay in initiating effective therapy, high risk of mortality, and high risk of ESRD explain the urgent need for AKI biomarkers that could be detected early and noninvasively. Furthermore, identification biomarkers may aid to understanding the mechanism underneath this enigmatic condition. Numbers of studies have been performed using urine proteomics that are reviewed as follows (Table 5).

Nguyen et al. performed a proteomic study on urine samples of sixty patients undergoing cardiopulmonary bypass (CPB) and investigated acute renal injury in this cohort [109]. Urine protein profile of these patients obtained by SELDI-TOF-MS and biomarkers with* m/z* of 28.5, 43, and 66 kDa were suggested for prediction of AKI at 2 h following CPB with sensitivity and specificity of 100%. This study had promising results and highlighted the potential application of SELDI-TOF in screening patients with AKI risk. Further investigation by this group with proteomic approaches revealed aprotinin as a predictor of AKI, 2 h after initiation of CPB with sensitivity of 92% and specificity of 96% [110]. Metzger et al. performed CE-MS analysis to identify peptides predictive of AKI [111]. They suggested 20 urinary polypeptides specific for AKI and validated them in two independent, blinded ICU and cross-sectional HSCT patient cohorts. In addition, their suggested markers could detect AKI accurately 5 days before the rise of serum creatinine. The data showed overrepresentation of peptides of albumin, *α*-1-antitrypsin, and *β*-2-microglobulin and underrepresentation of fibrinogen *α* and collagens 1*α*(I) and 1*α*(III) fragments with accuracy of 91%.

A comprehensive study in terms of study design was performed by Aregger et al. on patients undergoing elective cardiopulmonary bypass [112]. They analyzed protein profile in spot urine samples from thirty-six patients before and after CPB and investigated AKI (according to RIFLE criteria) using 2D-DIGE and MALDI-TOF-MS. Regulated proteins in comparison between patients before and after CPB were inflammation-associated or tubular dysfunction-associated proteins while modified urinary albumin, zinc-alpha-2-glycoprotein (ZAG), and a fragment of adrenomedullin-binding protein were associated with AKI. An independent cohort of 23 patients with and 45 patients without acute kidney injury was further examined for ZAG (as it was nonmodified and nonfragmented) using western blot and ELISA. The positive points of their study were validation in an independent cohort by two methods (WB and ELISA) and definition of comprehensive exclusion criteria including great amount of proteinuria and microhematuria, CKD patients with low GFR, use of radiocontrast media or nonsteroid anti-inflammatory drugs (NSAIDs), and need for urgent surgery.

Further investigation of urinary biomarkers was performed later, on the larger cohort using similar method by this research group. Sixty-four critically ill patients of whom 52 had AKI were analyzed by 2D-DIGE for separation followed by LC-ESI-MS/MS for identification of differential spots [113]. In this study, *α*-1 microglobulin, *α*-1 antitrypsin, apolipoprotein D, calreticulin, cathepsin D, CD59, insulin-like growth factor-binding protein 7 (IGFBP-7), and neutrophil gelatinase-associated lipocalin (NGAL) were suggested as candidate markers. IGFBP-7 and NGAL were selected for further validation using ELISA in an independent verification group of 28 patients with and 12 control patients without AKI. IGFBP-7 had better accuracy for prediction of renal outcome in their cohort.

In the most recent study, Bell et al. have investigated the association of nonrenal factors with elevated biomarker levels in AKI using ELISA [114]. They reported NGAL and cystatin C, two famous biomarkers of AKI, as well as urinary [TIMP-2]·[IGFBP7] as poor predictors which could not predict AKI within 12 to 48 hours and might be affected by factors other than AKI. This finding indicates the need for more study of AKI predictive biomarkers despite large number of studies performed so far and the need for more practical and precise study design.

## 8. Specific Urinary Proteome Database

Identification and characterization of candidate biomarkers which were carefully extracted from bunch of complex and confusing data using appropriate statistical methods are a critical step in a typical study for biomarker discovery. Identification of the most robust urinary protein markers is enhanced by means of databases specific for urine and kidney proteins.

Currently there are a few available databases specific for human urine proteome. A number of urine databases are based on identified proteins derived from tryptic peptides of which MAPU [115] and Sys-BodyFluid [116] are more stated. The other useful urine specific databases are tabulated in Table 6.

Max Planck Institute has provided a proteome database entitled MAPU which consists of different sources such as tear, urine, seminal fluid, and tissue from* Homo sapiens* and* Mus musculus* [115]. In the urine part MAPU contains information about 1543 proteins which were separated and fractionated using one-dimensional SDS-PAGE and reverse phase HPLC and analyzed with the LTQ-FT and LTQ-Orbitrap at p.p.m. accuracy after both in-gel and in-solution digestion. It worth to note that approximately half of these deposited proteins are membrane proteins according to gene ontology (GO) analysis.

Sys-BodyFluid is a comprehensive proteome database which is composed of 11 body fluid proteomes including urine [116]. The data deposited in this database come from 50 peer-review publications of different laboratories across the world. Information and annotation of proteins are description, gene ontology, domain information, protein sequence, and involved pathway.

Mosaiques diagnostics database is known as peptidome urinary database including 13027 urine samples taken from both diseased and healthy subjects that is obtained by analytical platform CE-MS [117]. Another part of the urinary Mosaiques database is biomarker sequence information which was obtained for 953 peptides that are deriving from 116 different proteins [115, 117].

Human urinary proteomic fingerprint database (UPdb) was established in 2013, using urine samples from 200 individuals analyzed by SELDI-MS on several chip surfaces (SEND, HP50, NP20, Q10, CM10, and IMAC30). The database lists 2490 unique peaks/ion species from 1172 nonredundant SELDI analyses as well as 1384 included peaks from external studies using CE-MS, MALDI, and CE-MALDI hybrids. The database provides information relating to the MS environment, subfractionation methods, chromatography setups, studied diseases, identified biomarker, statistical information, and identified proteins.

## 9. Conclusion

Urine is a valuable source of molecules which is capable of being diagnostic markers specially for renal diseases. The strength of urine in comparison to plasma and tissue samples is the noninvasive collection procedure and less complex protein content. The complications in biopsy based diagnosis (i.e., invasiveness, dependence of diagnosis on pathologist tact and observation, limitations due to infection, hypertension, and kidney size) make urinary biomarkers a safe reliable complement alternative way for diagnosis beside traditional biopsy. In addition, lack of limitation in amount of specimen at the time of collection and relatively stable content of peptides and proteins because of complete proteolytic process by endogenous proteases during the storage in the bladder make urine an ideal specimen for biomarker researches. As urinary proteins are mainly originating from kidney tissue (~70%) (remaining 30% derived from plasma), therefore urine is the most appropriate sample for biomarker discovery in renal disease, urogenital track, and vascular system.

However, molecular biomarkers have not become practical in clinics yet, and extensive attempts have been devoted to validate these molecular markers. Proteomic techniques beside advanced statistical analysis and bioinformatics knowledge are versatile tools in urinary biomarker discovery. It is expected that advances in analytical tools and software programs as well as accurate study design in the near future will improve sensitivity and specificity of available biomarkers.

## Figures and Tables

**Table 1:** Putative biomarkers for diabetic nephropathy discovered in urine proteome analysis.

Protein biomarkers	Up/downregulation	Cohort	Technique	Type of biomarker	Reference
Pancreatic amylase deoxyribonuclease I	↓	Healthy controls (*n* = 27) and diabetic patients (*n* = 27)	8-plex iTRAQ	Diagnostic	[80]

CD36	↑	Type 2 diabetes (T2DM) with normoalbuminuria (*n* = 20) and T2DM with microalbuminuria (*n* = 20) and T2DM with macroalbuminuria (*n* = 20)	ELISA	Prognostic	[118]

Podocyte-secreted Angptl4	↑	Diabetic rats	ELISA	diagnostic and therapeutic biomarker	[119]

Gelsolin and antithrombin-III			GeLC-MS/MS	Diagnostic	[120]

Ephrin type-B receptor 4 and vitamin K-dependent protein Z			GeLC-MS/MS	Prognostic	[120]

AMBP, MLL3, and VDAC1			LC-MS/MS	Diagnostic	[121]

Fetuin-A	↑	DN patients (*n* = 85)	Lectin microarray	Prognostic	[122]

Collagen fragments (specially collagen *α*-1(I) chain)	↓	DN patients (*n* = 35)	CE-MS	Diagnostic	[79]

**Table 2:** Putative biomarkers for IgAN discovered by urine proteome analysis.

Protein biomarkers	Up/downregulation	Cohort	Technique	Type of biomarker	Reference
GP2, vasorin, and EGF	↓	Healthy controls (*n* = 8) and gAN patients (*n* = 13)	Nano-LC-MS/MS	Diagnostic	[86]

CLM9, protocadherin, uteroglobin, DDPIV, NHLC3, and SLAF5	↑	Healthy controls (*n* = 8) and IgAN patients (*n* = 13)	Nano-LC-MS/MS	Diagnostic	[86]

MBL	↑	IgAN patients (*n* = 62) and control (*n* = 50)	ELISA	Diagnostic	[87]

sTfR	↑	IgAN patients (*n* = 71) and control (*n* = 50)	Latex-enhanced immunonephelometric assay	Diagnostic	[88]

Albumin fragments, *α*-1-antitrypsin, and *α*-1-*β*- glycoprotein	↑	IgAN patients (*n* = 43) and control (*n* = 50)	2DE-MALDI-TOF-TOF and ELISA	Prognostic	[123]

LG3	↓	IgAN patients (*n* = 43) and control (*n* = 50)	2DE-MALDI-TOF-TOF and ELISA	Diagnostic	[123]

**Table 3:** Putative biomarkers for focal segmental glomerulosclerosis discovered in urine proteome analysis.

Protein biomarkers	Up/downregulation	Cohort	Technique	Type of biomarker	Reference
Collagen IV, cadherin	↓	AD-treated mice (*n* = 6) and normal control (*n* = 6)	2DE-MS validated by western blot	Prognostic	[90]

Glutathione S-transferase	↑		2DE-MS validated by western blot	Prognostic	[90]

ADAM32, Cerberus, apoptosis-inducing factor-2, and Annexin A1	↑			Diagnostic	[90]

Tomoregulin	↓			Diagnostic	[90]

Albumin, serotransferrin, alpha-1-antiproteinase, afamin, ceruloplasmin, plasminogen, and AMBP	↑	AD-treated rat (*n* = 13)	LC-MS/MS validated by western blot	Prognostic	[92]

ApoA-Ib	↑	Relapsing group (*n* = 8), nonrelapsing group (*n* = 27) for test and relapsing group (*n* = 6), nonrelapsing group (*n* = 34) for validation	2DE-MALDI-TOF-MS and LC-MS/MS validated by western blot	Predictive	[124]

TRFE, A1AT, ApoA-1, ANT3, A1AG1, and Robo4	↑	FSGS (*n* = 11), IgAN (*n* = 6), and healthy control (*n* = 8)	nLC-MS/MS	Diagnostic	[71]

CD59, CD44, IBP7, UROM, GRN, and SAP	↓			Diagnostic	[71]

DPEP1	—			Diagnostic	[71]

Haptoglobin	↓	Mild disease state (*n* = 5), advance disease state (*n* = 5)	nLC-MS/MS	Prognostic	[70]

Ribonuclease 2	↑			Prognostic	[70]

APOA-1	↑	Steroid sensitive FSGS (*n* = 6), steroid resistant FSGS (*n* = 4)	nLC-MS/MS	Predictive	[69]

MXRA8	↓			Predictive	[69]

13.8 kDa A1BG fragment	↑	Steroid resistant nephrotic syndrome (*n* = 19), steroid sensitive nephrotic syndrome (*n* = 15), and controls (*n* = 10)	SELDI-TOF-MS	Predictive	[125]

**Table 4:** Biomarker candidates for MN discovered by urine proteome analysis.

Protein biomarkers	Up/downregulation	Cohort	Technique	Type of biomarker	Reference
VEGF	↓	MN (*n* = 30), minimal change disease (MCD) (*n* = 8), FSGS (*n* = 10), necrotizing glomerulonephritis (*n* = 8), DN (*n* = 12), and healthy control (*n* = 33)	ELISA	Diagnostic	[126]

*β*2m	↑	iMN (*n* = 57)	ELISA	Prognostic-predictive	[127]

Nephrin	—	Passive Heymann nephritis (*n* = 16), normal rat (*n* = 5)	Western blot	Qualitative diagnostic	[128]

LIMP-2	↑	iMN (*n* = 5), FSGS (*n* = 5), and healthy control (*n* = 3)	iTRAQ and verification with immunofluorescence of glomeruli	Diagnostic	[104]

*α*1M, URBP	—	Complete remission (CR) (*n* = 1), partial remission (PR) (*n* = 9), limited response (LR) (*n* = 5), and nonresponders (NR) (*n* = 4)	Nephelometry	Predictive	[106]

**Table 5:** Urinary candidate biomarkers for AKI.

Protein biomarkers	Up/downregulation	Cohort	Technique	Type of biomarker	Reference
IL-18	↑	AKI after CBP (*n* = 20), controls (*n* = 35)	ELISA	Predictive	[129]

KIM-1, NAG, and NGAL	↑	AKI (*n* = 36), early AKI (*n* = 16), late AKI (*n* = 20), and non-AKI (*n* = 54)	ELISA	Predictive	[130]

Aprotinin	↑	106 pediatric patients undergoing CPB	SELDI-TOF-MS	Predictive	[110]

NGAL	↑	71 children undergoing cardiopulmonary bypass	Western blot and ELISA	Predictive	[131]

KIM-1	↑	Cisplatin-induced nephrotoxic rats (*n* = 4)	ELISA	Predictive	[132]

Albumin, *α*-1-antitrypsin, and *β*-2-microglobulin	↑	AKI (*n* = 16) and non-AKI (*n* = 14) from ICU patients as training set, AKI (*n* = 9) and non-AKI (*n* = 11) from ICU patients, and AKI (*n* = 13) and non-AKI (*n* = 19) from HSCT patients as validation set	CE-MS	Diagnostic	[111]

Fibrinogen *α* and collagens 1*α*(I) and 1*α*(III)	↓		CE-MS	Diagnostic	[111]

Zinc-alpha-2-glycoprotein and a fragment of adrenomedullin-binding protein	↓	Discovery cohort: AKI (*n* = 6) and non-AKI (*n* = 6); validation cohort: AKI (*n* = 23) and non-AKI (*n* = 45)	2D-DIGE and MALDI-TOF-MS and validation by ELISA and western blot	Predictive	[112]

IGFBP-7 and NGAL	↑	LNR (*n* = 26), ER (*n* = 26), and control (*n* = 12)	2D-DIGE and validation by ELISA	Diagnostic/prognostic	[113]

Netrin-1	↑	AKI (*n* = 26), non-AKI (*n* = 34)	ELISA	Predictive	[133]

*α*1-microglobulin, *α*1-acid glycoprotein, and albumin	↑	Discovery cohort: AKI (*n* = 15) and control (*n* = 15); validation cohort: AKI (*n* = 135) and control (*n* = 230)	SELDI-TOF-MS and validation by nephelometry	Diagnostic/prognostic	[134]

Hsp72	↑	AKI (*n* = 17), control (*n* = 20)	ELISA	Predictive	[135]

**Table 6:** Databases for proteomics of urine.

Database	Organization/company	Address
Sys-BodyFluid	Shanghai Institutes for Biological Science	http://www.biosino.org/bodyfluid/

MAPU	Max Planck Institute for Biochemistry (Germany)	http://www.mapuproteome.com/

HKUPP	Human Proteome Organization	http://www.hkupp.org/

Urinary Exosome Protein Database	NHLBI Laboratory of Kidney and Electrolyte Metabolism	http://dir.nhlbi.nih.gov/papers/lkem/exosome/index.htm

Urinary Protein Biomarker Database	Chinese Academy of Medical Sciences and Peking Union Medical College	http://122.70.220.102/biomarker/

Mosaique	The Mosaiques Diagnostics & Therapeutics AG (Germany)	http://mosaiques-diagnostics.de/diapatpcms/ mosaiquescms/front_content.php?idcat=257

## Abbreviations

IgA: Immunoglobulin A
2D: Two-dimensional
2DE-MS: Two-dimensional gel electrophoresis followed by mass spectrometry
LC-MS: Liquid chromatography coupled to mass spectrometry
CE-MS: Capillary electrophoresis coupled to mass spectrometry
2D-DIGE: Two-dimensional difference gel electrophoresis
LC: Liquid chromatography
SCX: Strong cation exchange column
FTICR: Fourier transform ion cyclotron resonance
SELDI-TOF: Surface-enhanced laser desorption/ionization time of flight
DN: Diabetic nephropathy
MA: Microalbuminuria
THP: Tamm-Horse fall urinary glycoprotein
ZA2G: Zinc-*α*-2 glycoprotein
IgAN: IgA nephropathy
IGFBP7: Insulin-like growth factor-binding protein 7
TEC: Tubular epithelial cell
LN: Lupus nephritis
SLE: Systemic lupus erythematous
MCP-1: Monocyte chemotactic protein
AGP: *α*1-acid-glycoprotein
LPDGS: Lipocalin-type prostaglandin D-synthetase
CVD: Cardiovascular disease
CAD: Coronary artery disease
HF: Heart failure
NCD: Noncardiac dyspnea patients
CHF: Chronic heart failure
AHF: Acute heart failure
IGFBP2: Insulin-like growth factor binding protein 2
NF-*κ*B: Nuclear factor *κ*B
IBS: Irritable bowel syndrome
GI: Gastrointestinal
TFF3: Trefoil Factor 3
uGI: Upper gastrointestinal
GA: Genetic algorithm
FDP: Fibrinogen degradation products
GO: Gene ontology
UPdb: Urinary proteomic fingerprint database
LNR: Late/nonrecovery
ER: Early recovery
AKI: Acute kidney injury
MN: Membranous nephropathy
iMN: Idiopathic membranous nephropathy
PHN: Passive Heymann nephritis
FSGS: Focal segmental glomerulosclerosis
iTRAQ: Isobaric tags for relative and absolute quantification
LIMP-2: Lysosome membrane protein-2
RP: Reverse phase
U*α*1M: Urinary alpha 1 microglobulin
URBP: Urinary retinol binding protein
ICU: Intensive care unit
CPB: Cardiopulmonary bypass
WB: Western blot
CKD: Chronic kidney disease
NSAIDs: Nonsteroid anti-inflammatory drugs
IGFBP-7: Insulin-like growth factor-binding protein 7
NGAL: Neutrophil gelatinase-associated lipocalin
ADR: Adriamycin
MARS: Multiple affinity removal system
FSGS: Focal segmental glomerulosclerosis.
